# Alcohol and Insanity

**Published:** 1909-12-18

**Authors:** 


### Alcohol and Insanity.

Dr. W. A. Parker, the Medical Superintendent of the Glasgow District Mental Hospital, in his annual report for 1908-9 deals at some length with the question of alcohol as a factor in the causation of insanity. The subject is far too broad to be discussed adequately within the limits of an annual report, and the statistics adduced, however interesting, are hardly convincing. "Alcohol alone or in combination," remarks Dr. Parker, "is the most prolific determining cause of the insanity in our admissions. It is credited with being the immediate cause in forty-seven cases, and in combination with other causes in other eighteen cases; sixty-five in all, or 23 per cent. of the admissions. Besides this direct action, drunkenness is as notable a feature as ever among the parents of those of our admissions, whose break-down or arrest of mental growth has taken place during the period of most rapid development. Out of two hundred and eighty-two admissions there were one hundred and seventy-two in whose cases no accurate information could be got on the point of the parents' habits regarding alcohol. In one hundred and ten cases admitted a definite history was obtained on this point. Of these one hundred and ten a history of parental drunkenness was got in fifty-nine instances, and in fifty-one the parents of the patients admitted were sober or total abstainers. This gives a percentage of 53.6 of the cases admitted showing a history of parental abuse of alcohol; but if the cases in which the first mental illness occurred before the end of the twenty-sixth year be separated from the rest we find that in the adolescent cases 78.8 per cent. show a history of parental alcoholism; while of the others only 40 per cent. do so. It should be noted that these figures really understate the case." There is no doubt that a history of abuse of alcoholic drinks is frequently to be obtained in the majority of cases of insanity, but it is still to be convincingly shown that the abuse of alcohol is a causative factor in mental disease, any more than the action of sulphuric acid in the air is the causative factor in cancer. Statistics are wonderful things, but it is not everyone, and least of all the medical man, who can handle them fairly. For that reason we are loth to accept such generalisations as the above. Were it possible to collate all the available statistics and present them in bulk, leaving anyone who is interested in the matter to draw his own conclusions from their testimony, the case against alcohol might be much strengthened. Even then it would need very careful and scrupulously impartial compilation of medical, personal, and family history in each case. The subject is one of more than ordinary difficulty, especially if it is desired to settle the question so ably supported by Dr. Reid that the alcoholic tendency is inherited. Questions of heredity *versus* habit, inborn propensities *versus* propensities derived from the influence of environment or example, are very hard to settle. We need here only allude to the serious work that has lately been done in Germany and Italy on the subject of the relation of alcoholism to sexual perversion to show how manifestly absurd it is to take the testimony of isolated statistical tables as conclusive. Theorising on a large scale is very effective, especially when one has an audience of total prohibition fanatics to deal with; but sensible temperance reformers will not feel justified in accepting all the conclusions so far published on the subject of alcohol as a causative factor in mental or other diseases as universally applicable until far more evidence than is at present available is forthcoming.

